# Atomistic insight into the minimum wear depth of Cu(111) surface

**DOI:** 10.1186/1556-276X-8-514

**Published:** 2013-12-05

**Authors:** Zengqiang Li, Yanhua Huang, Junjie Zhang, Yongda Yan, Tao Sun

**Affiliations:** 1 Center for Precision Engineering, Harbin Institute of Technology, Harbin, 150001, People’s Republic of China; 2 Research Center of Laser Fusion, China Academy of Engineering Physics, Mianyang 621900, People’s Republic of China

**Keywords:** Single asperity friction, Friction and wear, Single crystalline Cu, Incipient plasticity, Molecular dynamics

## Abstract

In the present work, we investigate the minimum wear depth of single crystalline Cu(111) under single asperity friction by means of molecular dynamics simulations. The atomistic mechanisms governing the incipient plasticity are elucidated by characterizing specific defect structures and are correlated to the observed mechanical and frictional responses of the material. Furthermore, the effect of probe radius on the friction process is studied. Our simulations indicate that the formation of wear impression is closely associated with defect nucleation and the minimum wear depth is equivalent to the critical penetration depth at which plasticity initiates. It is found that the probe radius has a strong influence on the formation of defect structures and the observed mechanical responses.

## Background

With the feature size of miniaturized mechanical components shrinking down to the nanometer regime, friction and wear, as the major causes of mechanical failures and dissipative energy losses, play pronounced and even dominant role in determining the functionality of nanoelectromechanical system (NEMS) devices [1-3]. Therefore, reducing the friction and wear between contacting surfaces of components is of significant importance for the application of NEMS devices. Specifically, a fundamental understanding of the atomic scale origin of the friction-induced wear is essentially required for the rational design of the components that possess good wear resistance.

During the course of friction, wear phenomena are closely accompanied with permanent deformation and even removal of the materials under applied mechanical loads. Thus, identifying and characterizing the initiation of plasticity of the materials under friction are central to the understanding of the atomic scale origin of wear phenomena. In the past few decades, both experimental investigations and atomistic simulations have been conducted to investigate the incipient plasticity of metallic and semiconductor materials under nanoindentation [4-8]. Recently, Paul et al. performed nanoindentation experiments to study the minimum threshold of the incipient plasticity of a gold single crystal. They found that the indentation-induced elastic deformation and plastic deformation can be well identified by features observed in the force-displacement curves, and the first pop-in phenomenon reflects the onset of plasticity [9]. However, a rather limited effort has been taken to study the incipient plasticity which occurs under friction. Compared to the localized uniaxial stress state of nanoindentation, the multi-axial states of localized stress induced by friction action may lead to more complex mechanical responses at the onset of plasticity. On the other hand, it is crucial to correlate microstructure evolution that occurs within the materials with the observed features in force-displacement curves, which is of great challenge for the experimental investigations because of the involvement of nanometer length scale. As a complement to experiments, molecular dynamics (MD) simulation has been demonstrated to be one powerful tool to investigate the atomic scale phenomena of friction and wear [10-20]. Although previous MD simulations have provided valuable insights into the nanoscale friction and wear processes, our knowledge about the incipient plasticity under friction process, particularly the relationship between specific defect structures and observed wear phenomena, is still scarce.

In the present work, we perform MD simulations to investigate the incipient plasticity of single crystalline copper under single asperity friction with a spherical probe. The deformation mechanisms of the material are analyzed in detail, and the specific defect structures are particularly characterized and are correlated to the mechanical and frictional responses. Our simulations demonstrate that the minimum wear depth is determined by the formation of permanent defects such as dislocations and vacancies and is strongly probe radius-dependent. This paper is outlined as follows. In ‘Methods’ Section, we describe the simulation method. The simulation results are presented and discussed in Section ‘Results and discussion’. Finally, we summarize the results in ‘Conclusions’ Section.

## Methods

**Figure 1:**
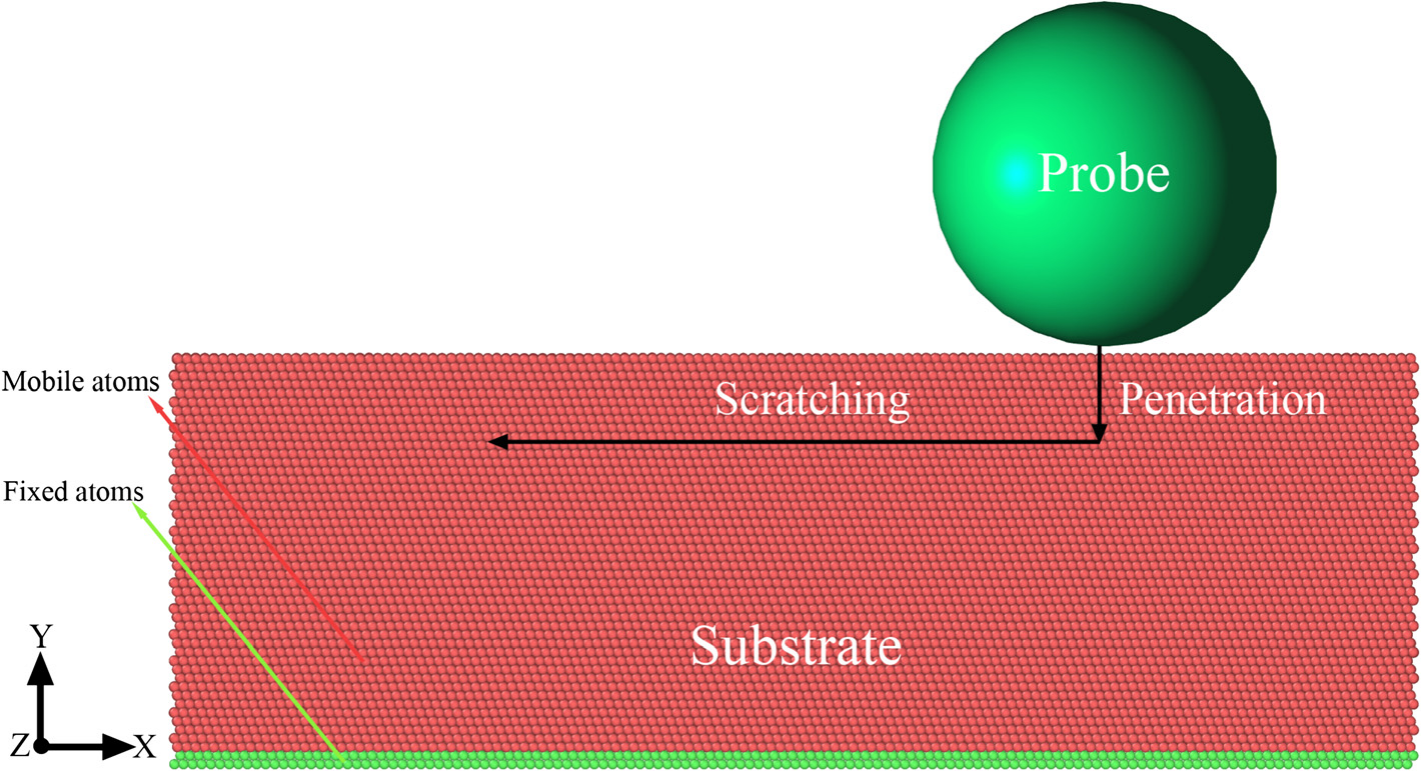
**MD model of single asperity friction of single crystalline copper.** The atoms in the substrate are colored according to their virtual types, as red for mobile atoms and green for fixed atoms.

As depicted in Figure 1, the MD model of single asperity friction employed in the present work consists of a substrate and a spherical probe. The substrate of single crystalline copper has a dimension of 30, 10, and 30 nm in *X*[2], *Y* [111], and *Z*[1-10] directions, respectively. Periodic boundary conditions are imposed in the transverse *X* and *Z* directions of the substrate. Figure 1 shows that the substrate is composed of two virtual types of atoms, as the green color stands for the fixed atoms and the red one represents the mobile atoms in which motions follow the Newton’s second law of motion. The atomic interactions within the substrate are described by an embedded atom method developed for copper [21]. The frictionless spherical probe is modeled by a strong repulsive potential [22]. To study the influence of probe radius on the friction, four probe radiuses of 6, 8, 10, and 12 nm are considered.

The atoms in the as-created substrate first undergo global energy minimization at 0 K, and then the substrate is relaxed to its equilibrium configuration at 30 K and 0 bar through dynamic NPT relaxation for 50 ps. After relaxation, the substrate is subjected to friction by placing the probe above the free surface of the substrate with a distance of 0.2 nm. The friction process is composed of two stages of first penetration and following scratching, as illustrated in Figure 1. In the penetration stage, the probe moves along negative *Y* direction with constant velocity of 20 m/s to penetrate into the substrate until a pre-determined penetration depth is reached. In the following scratching stage, the probe scratches at 12.2 nm along negative *X* direction with constant velocity of 20 m/s. Both the penetration and scratching velocities of 20 m/s are a few orders of magnitude higher than the typical velocities utilized in nanoscratching experiments due to the intrinsic requirement of integration timesteps to be of the order of 1 fs. All the MD simulations are completed using the IMD code with a time step of 1 fs [23]. The detailed description about the friction procedure can also be found elsewhere [24]. To identify the defects generated within the substrate, a modified bond angle distribution (BAD) method is utilized [25]. In the present work, the perfect face-centered cubic (FCC) atoms are not shown for better viewing of the defect structures, and the coloring scheme for various defects is as follows: red stands for surface atoms, blue indicates hexagonal close-packed (HCP) atoms, and the remaining atoms are categorized into defects including dislocation cores and vacancies. The software AtomEye is employed to visualize MD data and generate MD snapshots [26].

## Results and discussion

### Determination of minimum wear depth

**Figure 2:**
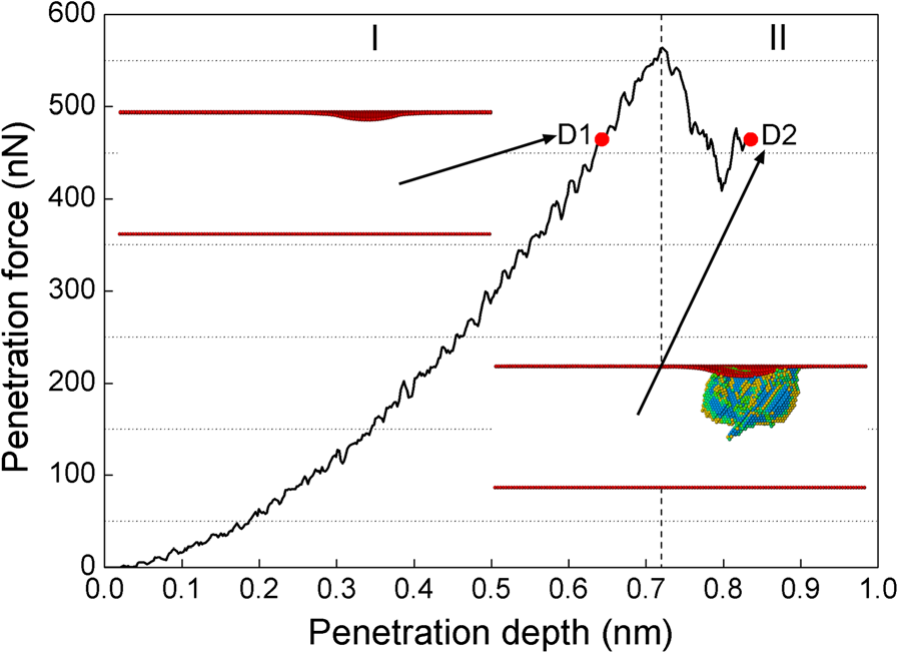
**Penetration force-penetration depth curve during the penetration with a probe radius of 8 nm.** The two penetration depths D1 of 0.65 nm and D2 of 0.82 nm have the same penetration force. The two insets show instantaneous defect structures at D1 and D2, in which atoms are colored according to their BAD values and FCC atoms are not shown.

In the friction process, there are three force components acting on the probe, as scratching force along *X* direction, penetration force along *Y* direction, and lateral force along *Z* direction, respectively. In the penetration stage, both scratching force and lateral force mainly fluctuate around constant value of 0 because the probe only applies uniaxial localized stress along *Y* direction. Figure 2 plots the penetration force-penetration depth curve during the penetration stage with a probe radius of 8 nm, indicating that the deformation behavior of the substrate is divided into two regimes. In the regime I, the substrate undergoes elastic deformation, accompanied with rapid increase of the penetration force. After the penetration depth reaches a critical value of 0.72 nm, the penetration force drops precipitously, indicating the occurrence of elastic deformation-plastic deformation transition. The observed phenomenon of force drop, which corresponds to the pop-in event widely observed in the load-controlled nanoindentation experiments, is caused by dislocation avalanche beneath the penetrated surface [5,7,24]. We note that the tribochemistry, e.g., the presence of cupric oxide, may significantly alter the deformation behavior of the topmost surface. In the regime II, the substrate undergoes plastic deformation dominated by dislocation activities. The action of penetration stops at a penetration depth D2 of 0.82 nm. Another penetration depth D1 of 0.65 nm in the elastic deformation regime, at which the penetration force is equal to that at D2, is also marked in Figure 2. The two insets in Figure 2 present instantaneous defect structures obtained at the two penetration depths D1 and D2, respectively. While the substrate is purely elastically deformed at D1, there is a considerable amount of defects formed beneath the penetrated surface at D2.

**Figure 3:**
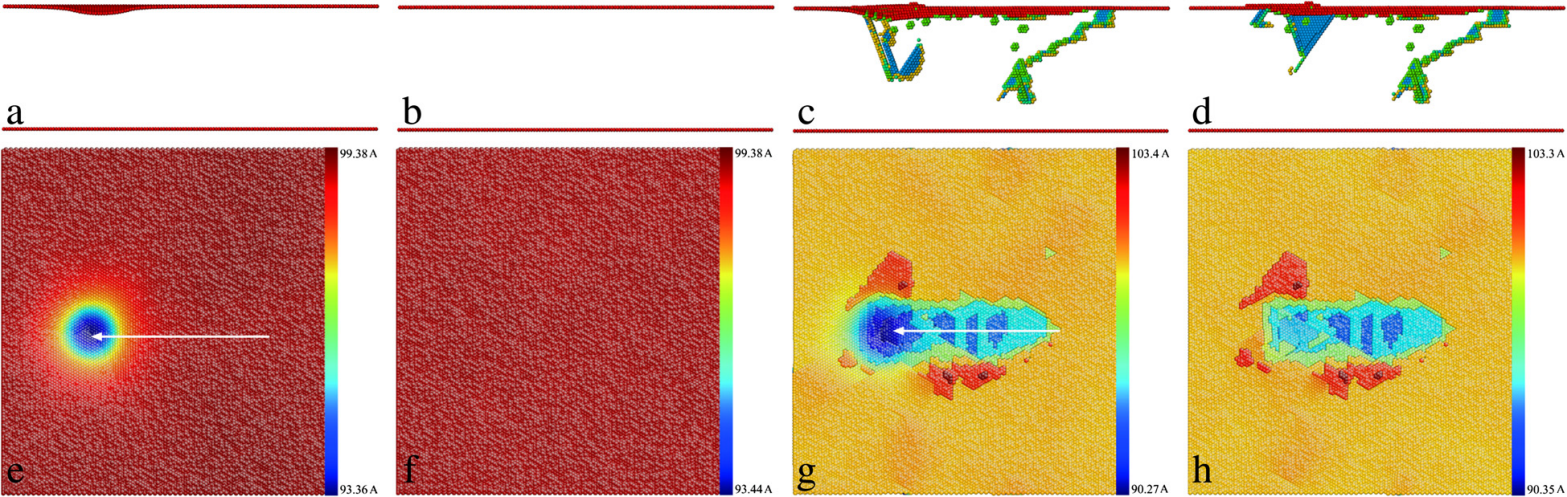
**Defect structures and surface morphologies after scratching and retraction under D1 and D2 (a,b,c,d).** Defect structures after scratching and retraction under the scratching depths D1 and D2, respectively. Atoms are colored according to their BAD values, and FCC atoms are not shown. **(e,f,g,h)** Surface morphologies after scratching and retraction under the scratching depths D1 and D2, respectively. Atoms are colored according to their heights in *Y* direction.

While Figure 2 shows that the defect structures at the two penetration depths are significantly different, two scratching simulations under the two scratching depths D1 and D2 are conducted with the same probe radius of 8 nm. Under the scratching depth D1, both the penetration force and scratching force remain constant values throughout the scratching stage. However, the scratching force is far smaller than the penetration force because of the absence of permanent deformation in the vicinity of the probe. We also note that the non-adhesion between the substrate and the probe in the current simulated system also contributes to the ultra-small scratching force. In contrast, under the scratching depth D2, the friction coefficient first increases rapidly and then fluctuates around constant value with large amplitudes when the scratching becomes stable. To examine the evolutions of defect structures and surface morphologies, retractions of the probe along *Y* direction to its initial height are conducted right after the completion of the two scratching stages. Figure 3 presents instantaneous defect structures and surface morphologies of the substrate after the completion of scratching and retraction for the two scratching depths. We note that the following observations are made based on not only the captured MD snapshots, but also the entire dynamic process provided by MD simulations: under the scratching depth D1, the substrate undergoes pure elastic deformation, and there is no defect formed beneath the surface after the completion of the scratching, as shown in Figure 3a. Accordingly, there is only one penetration impression formed on the surface shown in Figure 3e. Furthermore, Figure 3b,f demonstrates that the penetrated surface is fully recovered after the retraction, indicating that there is no permanent deformation that occurs within the substrate. Under the scratching depth D2, however, it is seen from Figure 3c that the defect zone beneath the surface extends significantly along the scratching direction. Figure 3g shows that there is one scratching-induced impression of the groove formed on the surface, and wear debris which accumulate on both sides of the groove is also observed. Although the penetrated surface undergoes tiny plastic recovery accompanied by the shrinking of the defect structures beneath the probe after the retraction, Figure 3d,h shows that both the defect structures, particularly those behind the probe, and the surface morphology are mainly unchanged. Furthermore, the height of wear debris increases slightly due to the annihilation of the dislocations at the surface [24].

**Figure 4:**
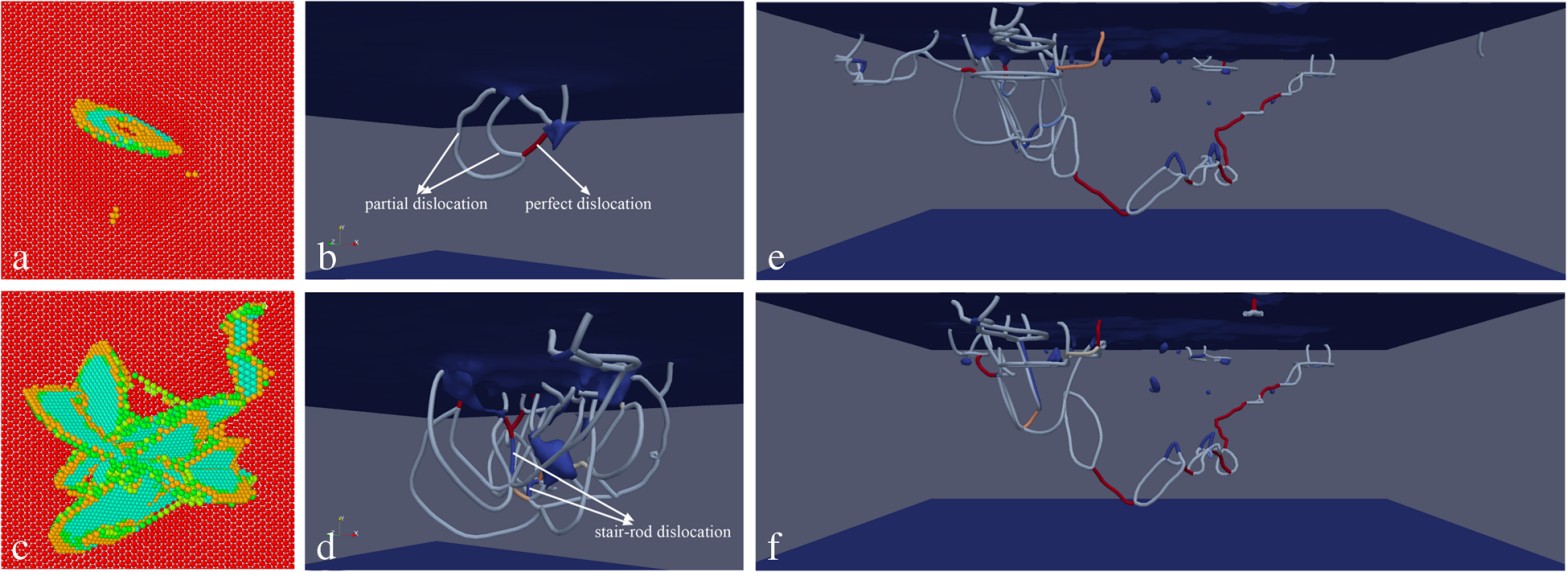
**Close inspections of defect structures in friction with a probe radius of 8 nm.** The scratching depth is 0.82 nm. **(a,c)** Bottom views of defect structures at penetration depths of 0.72 and 0.82 nm, respectively. Atoms are colored according to their BAD values and FCC atoms are not shown. **(b,d)** Dislocation networks shown in **(a)** and **(c)**, respectively. **(e,f)** Dislocation networks after the completion of scratching and retraction, respectively.

The above analysis indicates that the minimum wear depth is closely associated with the initiation of plasticity. To reveal the specific defect structures formed at the early stage of plastic deformation, a dynamic inspection of the defect evolution in the regime II of Figure 2 is performed. Figure 4a shows that at the critical penetration depth of 0.72 nm a dislocation loop formed on one {111} slip plane inclined to the (111) free surface, which leads to the sharp drop of the penetration force observed in Figure 2. The Burgers vector of each segment of the dislocation loop in Figure 4a is analyzed through DXA algorithm [27], and the corresponding dislocation network is presented in Figure 4b. Dislocation cores are represented by thin tubes, in which Shockley partial dislocation with 1/6 <112 > Burgers vector and perfect dislocation with 1/2 <110 > Burgers vector are colored gray and red, respectively. It is seen from Figure 4b that the dislocation loop consists of four partial dislocations and one perfect dislocation. In addition, there is one vacancy formed beneath the probe. Upon further penetration, the other three {111} slip planes are activated sequentially, and Figure 4c shows that the defect zone beneath the probe expands greatly. The glide of dislocations on adjacent slip planes leads to the formation of stair-rod dislocations with 1/6 <110 > Burgers vector highlighted by the arrows in Figure 4d. Figure 4e,f presents dislocation network after the completion of scratching and penetration, respectively. It is seen from Figure 4e that there is less dislocations but more vacancies in the wake of the probe than that in the vicinity of the probe due to the plastic recovery. In addition to the stair-rod dislocations, there are glissile prismatic dislocation loops formed by dislocation reaction and cross-slip events. In particular, the prismatic dislocation half-loops in front of the probe glide parallels to the free surface to transport the materials displaced by the probe without the formation of surface steps [24]. Although small part of the dislocations beneath the probe annihilates at the free surface during the retraction, Figure 4f shows that the defect structures are stable.

### Effect of probe radius on minimum wear depth

**Figure 5:**
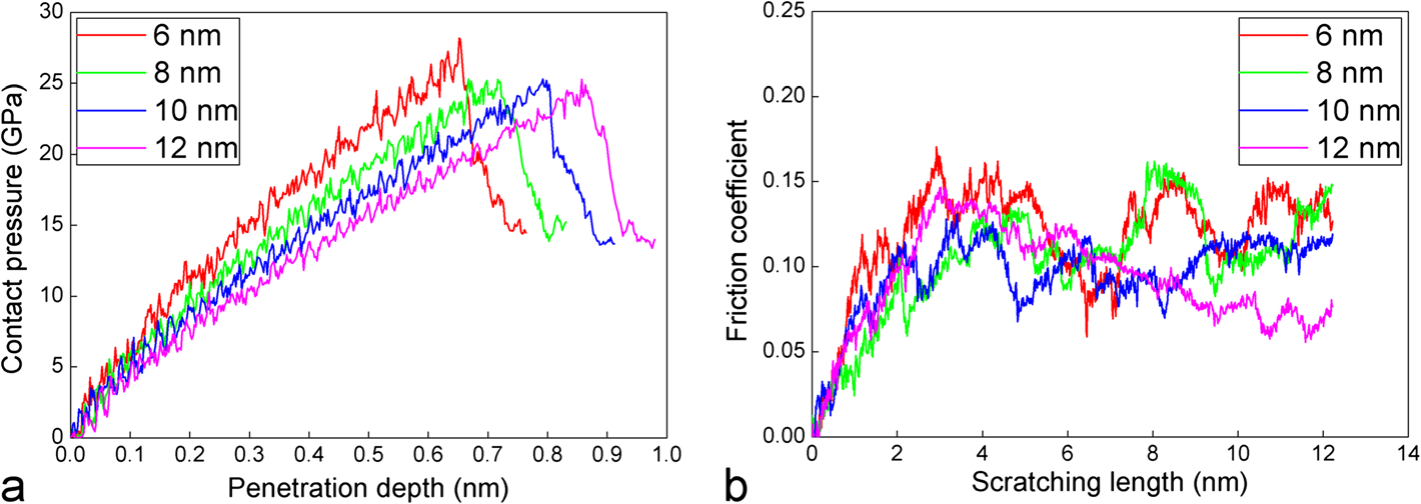
**Influence of probe radius on mechanical and frictional properties of the substrate under friction. (a)** Contact pressure-penetration depth curves. **(b)** Friction coefficient-scratching length curves.

To investigate the influence of probe radius on the minimum wear depth, friction simulations with another three probe radiuses of 6, 10, and 12 nm are conducted, in addition to the probe radius of 8 nm. For each probe radius, the penetration stage stops at a penetration depth that is 0.1 nm deeper than the critical penetration depth at which the phenomenon of force drop occurs. Figure 5a,b plots the contact pressure-penetration depth curves and the friction coefficient-scratching length curves during the penetration and scratching stages with the four probe radiuses, respectively. The contact pressure is defined as the ratio of the penetration force to the contact area. A detailed description about the calculation of the contact area during spherical penetration can be found elsewhere [28]. It should be noted that the maximum contact pressure shown in Figure 5a corresponds to the theoretical strength of the pristine and dislocation-free single-crystalline Cu. Furthermore, to quantitatively access the influence of probe radius on the frictional property of the substrate, the average friction coefficient is obtained by averaging more than 1,000 instantaneous points of friction coefficient in the range between 3 and 12.2 nm. Table 1 summarizes the mechanical responses of the substrate extracted during friction with the four probe radiuses. Figure 5a shows that the slope of the contact pressure-penetration depth curve in the elastic deformation regime decreases with increasing probe radius, indicating that the elastic deformation of the substrate is more compliant with the larger probe. However, the contact pressure reflecting the critical stress for initial dislocation nucleation from penetrated surface is approximately independent on the probe radius. It is seen from Table 1 that with the increase of the probe radius, both the critical force and the critical penetration depth associated with the initiation of plasticity increases, but the average friction coefficient decreases.

**Table 1:** Mechanical responses of the substrate under friction with different probe radiuses

**Probe radius**	**6 nm**	**8 nm**	**10 nm**	**12 nm**
Critical penetration force (nN)	387.1	565.9	814.4	1,081.1
Critical penetration depth (nm)	0.65	0.72	0.80	0.87
Critical contact pressure (GPa)	28.3	25.1	25.2	25.2
Average friction coefficient	0.126	0.118	0.103	0.098

**Figure 6:**
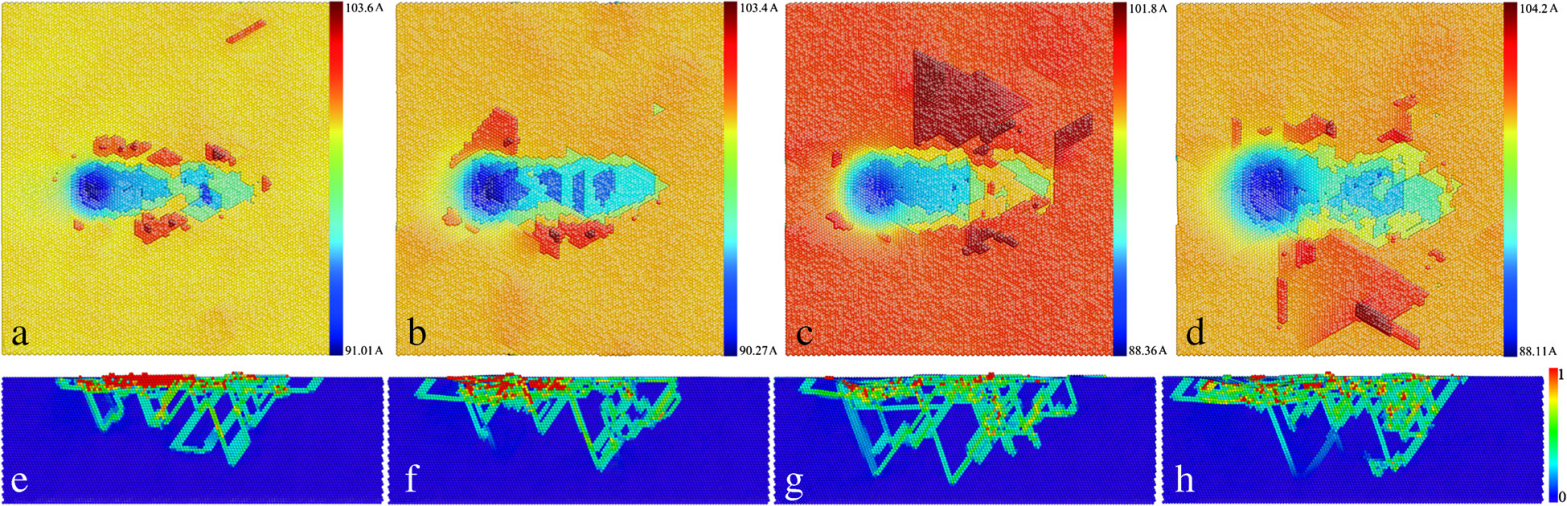
**Influence of probe radius on the friction of the substrate. (a,b,c,d)** Surface morphologies of the substrate after scratching with probe radiuses of 6, 8, 10, and 12 nm, respectively. Atoms are colored according to their height in *Y* direction. **(e,f,g,h)** Cross-sectional views of the substrate after scratching with probe radiuses of 6, 8, 10, and 12 nm, respectively. Atoms are colored according to shear strain ranging from 0 to 1.

Figure 6a,b,c,d presents the surface morphologies of the substrate after the completion of scratching with probe radiuses of 6, 8, 10, and 12 nm, respectively. A larger probe results in a larger volume and also wider extent of the wear debris, indicating that more atoms within the substrate are involved in the scratching action. To quantitatively characterize the scratching-induced motion of atoms, the shear strain of each atom is calculated by comparing the current atomic configuration of the substrate with the reference configuration obtained after relaxation. Figure 6e,f presents the cross-sectional views of the substrate after scratching with the four probe radiuses, respectively, in which atoms are colored according to their shear strains ranging from 0 to 1. It is seen from Figure 6 that the distributions of wear debris and shear strain are closely correlated for each probe radius. When probe radius is small, Figure 6e shows that the distribution of shear strain is compact and shallow. Furthermore, the atoms in the wear debris have significantly larger mobility than that within the material. In contrast, a lager probe leads to larger and more compliant distribution of shear strain.

**Figure 7:**
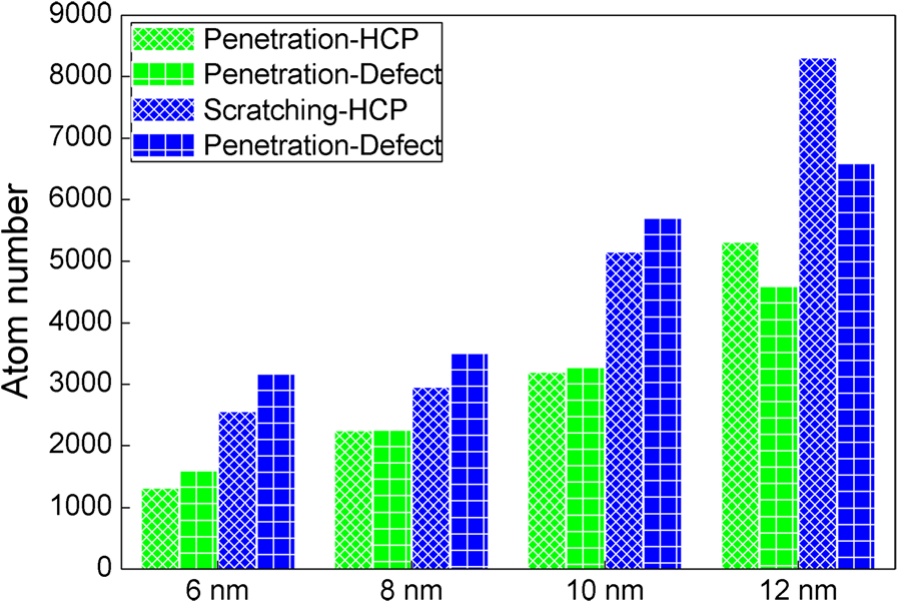
Influence of probe radius on numbers of HCP and defect atoms generated within the substrate under friction.

Figure 7 presents numbers of HCP and defect atoms generated within the substrate after penetration and scratching with the four probe radiuses. For each probe, there are more HCP and defect atoms generated in the scratching stage than that in penetration stage, because of the more complex plastic deformation associated with the multi-axial localized stress states. When the probe radius is not larger than 10 nm, there are more defect atoms than HCP atoms in both penetration and scratching stages for each probe radius. However, the friction with the probe radius of 12 nm results in more HCP atoms than defect atoms generated within the material. The formation of HCP atoms is associated with the activity of partial dislocations, while defect atoms are composed of not only dislocation cores but also vacancies. Therefore, Figure 7 indicates that the dislocation activity plays more pronounced role in governing incipient plasticity for larger probe. In addition, the incipient plasticity shows strong dependence on probe radius: the larger the probe, the larger both the HCP and defect atoms.

## Conclusions

In summary, we perform MD simulations to investigate the atomic scale origin of the minimum wear depth of single crystalline Cu(111) during single asperity friction. Simulation results show that scratching impression can only be made under a scratching depth at which there are permanent defects formed. It is indicated that the minimum wear depth is equivalent to the critical penetration depth associated with the first force-drop observed in the force-depth curve. The specific permanent defects governing the wear phenomena are composed of stair-rod dislocations and prismatic dislocation loops as well as vacancies. While the contact pressure for the nucleation of initial dislocation is independent on probe radius, the minimum wear depth increases with probe radius. Further analysis of the shear strain distribution implies that a larger probe results in more compliant deformation of the material, which leads to larger volume of wear debris and wider extent of defect structures.

## Competing interests

The authors declare that they have no competing interests.

## Authors’ contributions

JZ, YY, and TS conceived the project. LZ, YH, and JZ performed molecular dynamics simulations and analyzed data. LZ and JZ wrote the paper. All authors read and approved the final manuscript.
